# Changes of occlusal plane inclination after orthodontic treatment in different dentoskeletal frames

**DOI:** 10.1186/s40510-014-0041-1

**Published:** 2014-06-25

**Authors:** Jin-le Li, Chung How Kau, Min Wang

**Affiliations:** The State Key Laboratory of Oral Diseases, Department of Prosthodontics, West China School/Hospital of Stomatology, Sichuan University, Chengdu, 610041 PR, China; Department of Orthodontics, School of Dentistry, University of Alabama at Birmingham, Birmingham, 35294 AL USA

**Keywords:** Bisected occlusal plane, Functional occlusal plane, Maxillary occlusal plane, Mandibular occlusal plane, Inclination

## Abstract

**Background:**

The inclination of the occlusal plane (OP) is related to facial types and experiences physiological growth-related changes. The aims of this research were to determine if there were any differences in the inclination of OP in subjects with three types of skeletal malocclusion and to investigate the characteristics and differences of functional occlusal plane (FOP) compared to bisected occlusal plane (BOP).

**Methods:**

A sample of 90 Caucasians patients was skeletal-classified into three (*n* = 30), and pre- and post-treatment cephalograms were digitized. Six linear and 8 angular cephalometric measurements were selected. The changes of OP inclination within each group and the differences among the three groups pre- and post-treatment were compared with paired *t* test and ANOVA test, respectively. The comparison and correlation between BOP and FOP were analyzed with paired *t* test and coefficient of correlation, respectively.

**Results:**

The BOP angle increased in all of the three groups but only had statistically significant differences in skeletal class II patients in a mean of 1.51° (*p <* 0.05). The FOP-SN angle showed stability (*p >* 0.05) in all three groups. The inclination of FOP was closely related to that of BOP (*p <* 0.001) but revealed discrepancies in each group.

**Conclusions:**

BOP and FOP were statistically significantly steeper in class II subjects compared to the other two groups both before and after treatment. The BOP angle statistically significantly increased by 1.51° in skeletal class II patients. BOP was a more reproducible reference plane compared to FOP during cephalometric tracing process, while FOP showed stability in orthodontically treated patients with all three skeletal patterns.

**Electronic supplementary material:**

The online version of this article (doi:10.1186/s40510-014-0041-1) contains supplementary material, which is available to authorized users.

## Background

The importance of the occlusal plane (OP) in orthodontic has been especially stated in the literatures [1–4].

The form and inclination of the OP hold individual characteristics and are connected not only with the function of the stomatognathic system but also with the esthetics of dentofacial appearance. A functional correlation between the inclination of OP and the masticatory closing path has been observed. This is an important determinant in occlusion and one of the contributing factors to masticatory movement [5]. The upper smile arc is the relationship of the curvature of the maxillary incisal and canine edges to the curvature of the lower lip during the social smile, which is influenced by the OP angle. By producing a computerized prediction of the appearance of the smile at differing OP angles, Batwa et al. [6] concluded that changing the OP angle does affect relative smile attractiveness.

The cant of the posterior occlusal plane reflects the vertical height of occlusion, which is also associated with mandibular deviation in the same direction [7]. During dentoskeletal growth, reduced vertical height of dentition unilaterally affects the mandibular position, subsequently leading to a lateral condylar shift during functional movement, such as opening and closing. Occlusal deviations are related to transverse inclination of the OP, and contralateral differences in occlusal vertical dimension can reduce muscular balance eventually, resulting in a mandibular asymmetry [8].

The inclination of the maxillary posterior occlusal plane during growth and development can influence skeletal pattern and malocclusion type. There is potential differential maxillary and mandibular skeletal growth expressed along the OP [9]. The change in the inclination of the OP can alter the mandibular position relative to the maxillary occlusal surfaces as well as the condylar adaptive response to it, which plays a key role in the establishment of different dentoskeletal frames [2].

Before orthodontic treatment, patients’ teeth are in biomechanically neutral positions, which may or may not be correct from a functional or esthetic stand point. Orthodontic treatment changes the position and angulation of the teeth and moves them to an ideal esthetic and functional position. It is well recognized that small angular differences during orthodontic treatment can result in significant occlusion alterations [9], which could affect masticatory muscle balance [10, 11], cause functional disharmony and relapse [12].

The OP is a two-dimensional segmentation of a three-dimensional phenomenon; on cephalometric radiograph, a straight line is used to represent an imaginary plane at the level of occlusion. There are various ways for determining the occlusal plane, in which the bisected occlusal plane (BOP) is most commonly used. As proposed by Downs [13], the BOP is a line connecting the point bisecting the first molar cusp height and the point bisecting the incisal overbite. The functional occlusal plane (FOP) is a plane formed by bisecting the intercuspation of the first premolars and the intercuspation of the first molars [14, 15]. Maxillary and mandibular occlusal planes have also been used in literatures [16–18].

It has been stated that the inclination of the OP relates to facial types, with the class II facial types having a relatively steep angle, bending toward horizontal as the patient approaches class III [13]. Additionally, different orthodontic diagnosis and treatment plans could be made depending on the type of malocclusion and therapeutic goals, which might or might not change the OP [1, 19, 20]. Determination of OP is desirable to meet the demands of both esthetics [6] and function [5].

The goals of the present study are to:

- Cephalometrically evaluate OP inclination changes in orthodontically treated patients
- Compare the OP inclination in three different dentoskeletal frames before and after treatment
- Investigate the characteristics and differences of FOP compared to BOP

## Methods

### Sample description

Patients conforming to the American Board of Orthodontics (ABO) standards in the Orthodontics Department of the University of Alabama at Birmingham from 2009 to 2013 were selected. IRB approval was given by the University of Alabama at Birmingham to the study as a retrospective chart audit of finished cases in the department of orthodontics. The sample consisted of 43 male and 47 female Caucasian patients that commenced orthodontic treatment at mean age of 13.47 years and the mean active treatment time was 2.16 years. Table 1 shows group compositions.

**Table 1:** **Gro** **up data for pre-treatment ages and duration of treatment**

	Number	Female number	Male number	Pre-treatment age (years)	Duration of treatment
				Mean ± SD	Mean ± SD
Skeletal class I	30	14	16	13.75 ± 1.54	2.12 ± 0.50
Skeletal class II	30	17	13	13.03 ± 1.41	2.28 ± 0.49
Skeletal class III	30	16	14	13.63 ± 1.44	2.07 ± 0.48
Total	90	47	43	13.47 ± 1.48	2.16 ± 0.49

The sample was selected based on the following inclusion criteria: (1) adolescents with permanent dentition except third molars, (2) patients who finished treatment with non-extraction, (3) non-surgical cases, (4) complete and grade I lateral cephalogram images (high-quality image providing sufficient information with no errors from image taking procedure) both before and after treatment, (5) subjects who received comprehensive orthodontic treatment with fixed labial appliances in the upper and lower arches, (6) subjects who were treated only once (no re-treatments) and completed the treatment successfully. The exclusion criteria were patients older than 18 when the case started and subjects with anterior open bite.

#### Classification of subjects

Patients were classified skeletally based on the patient’s pre-treatment ANB angle:

Skeletal class I: 0**° <** ANB ≤ 4**°**

Skeletal class II: ANB > 4**°**

Skeletal class III: ANB ≤ 0**°**

The number of class III patients is limited and only 30 patients were included. In order to make the sample size of each group balance, we randomly choose 30 patients from class I and class II groups, respectively.

### Cephalometric analysis

The cephalograms from the pre- and post-treatment examination were taken with Orthoceph® OC100 D (Instrumentarium Corp., Helsinki, Finland) and imported into Dolphin Imaging software (Version 11.5; Dolphin Imaging & Management Solutions, Chatsworth, CA, USA). A custom cephalometric analysis was created and the landmarks used in this study are represented in Table 2. All landmarks were traced digitally by landmark identification and all measurements performed by Dolphin Imaging software. All cephalograms were traced and digitized by a single operator.

**Table 2:** **Variables measured from lateral cephalograms**

Variable	Description
Skeletal frame	
SNA°^a^	Angle formed by the SN plane and the Nasion-A point plane
SNB°	Angle formed by the SN plane and the Nasion-B point plane
ANB°	Angle between Nasion-A point plane and the Nasion-B point plane
MP-SN°	Angle formed by mandibular plane and SN plane
Occlusal plane	
BOP-SN°	Angle between the SN plane and BOP
FOP-SN°	Angle between the SN plane and FOP
MxOP-MnOP°	Angle between MxOP and MdOP
Dental	
Overbite (mm)	The vertical distance from U1 tip to L1 tip
Overjet (mm)	The horizontal distance from U1 tip to L1 tip
Maxillary dentoalveolar	
U1-SN°	The posterior-inferior angle formed by the long axis of the U1 and the SN plane
U1-PP (mm)	The perpendicular distance from the U1 tip to the palatal plane
U6-PP (mm)	The perpendicular distance from the U6 occlusal surface to the palatal plane
Mandibular dentoalveolar	
L1-SN°	The angle formed by the long axis of the lower central incisor and the mandibular plane
L1-MP°	The angle formed by the long axis of the lower central incisor and the mandibular plane
L1-MP (mm)	The perpendicular distance from the L1 tip to the mandibular plane
L6-MP (mm)	The perpendicular distance from the L6 occlusal surface to the mandibular plane

^a^ “°” represents the unit of angle measurement “degree”.

### Parametric and measurements

In this study, sella-nasion (SN) line was picked as the reference to study the change of inclination in the occlusal plane relative to the skeletal frame. This is because during general growth, the cant of SN line remains fairly unchanged [21]. Additionally, the sella and nasion have good accuracy and repeatability in lateral cephalograms. The angles SNA, SNB, ANB, and MP-SN were measured to evaluate the antero-posterior position of maxilla and mandible. The position and angulation of maxillary (U1) and mandibular central incisor (L1) and maxillary (U6) and mandibular first molar (L6) were measured from pre- and post-treatment lateral cephalograms to evaluate where the changes in the occlusal plane occurred.

**Figure 1:**
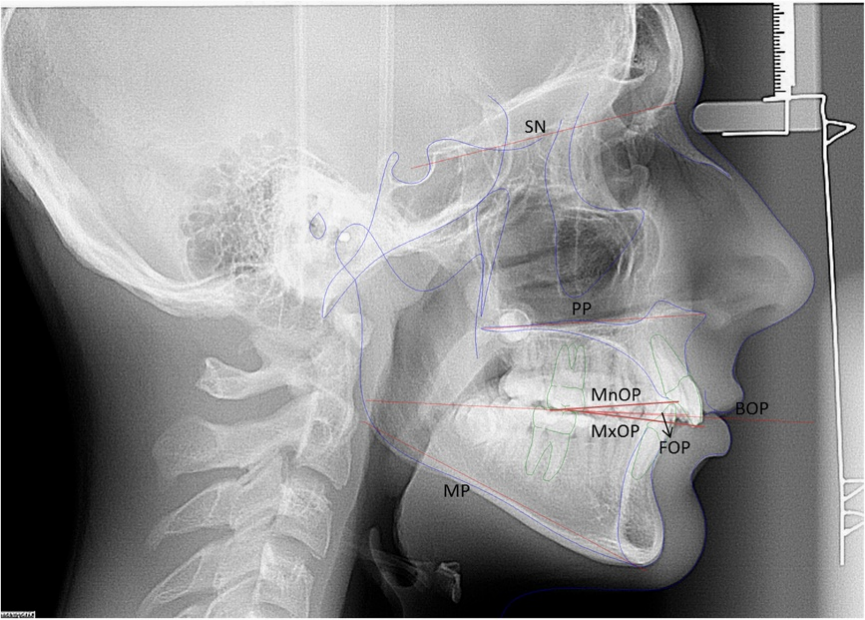
**Occlusal planes and reference planes used for cephalometric measurements.**

The planes used in this study (Figure 1) were defined as follows:

- Sella-nasion plane (SN), the line passing from the center of sella turcica to nasion
- Palatal plane (PP), the line defined by the anterior nasal spine and posterior nasal spine
- Mandibular plane (MP), the line joining gonion and menton
- Bisected occlusal plane (BOP), the line that bisects the vertical distance between the upper and lower incisal tips (U1 tip, L1 tip) and the upper and lower first molar occlusal surface (U6 occlusal, L6 occlusal)
- Functional occlusal plane (FOP), a line joining the point bisecting the U6 occlusal and L6 occlusal with the midpoint bisecting the intercuspation of the first premolars
- Maxillary occlusal plane (MxOP), a line drawn from the incisal edge of U1 to the midpoint of the U6 on the occlusal surface
- Mandibular occlusal plane (MnOP), a line drawn from the incisal edge of L1 to the midpoint of the U6 on the occlusal surface

The measurements related to occlusal plane and additional measurements are shown in Table 2.

### Assessing the method errors

Thirty cephalograms were chosen at random, and tracing was repeated by the same examiner 2 weeks after the first evaluation to test intra-examiner repeatability. Measurement errors were estimated using Dahlberg formula:

1 $$D=\sqrt{\sum_{i=1}^{N}\frac{d_{i}^{2}}{2N}}$$

where *d*_*i*_ is the difference between the first and the second measure and *N* is the sample size which was re-measured. The reliability coefficient values between the two timely separated readings were also calculated as recommended by Houston [22] to determine the reliability of all measurements.

Error of duplicate measurements was less than 0.4° for all angular measurements except SN-MP, BOP-SN, which were less than 0.6°, and FOP-SN which reached to 1.0°. The errors of all linear measurements were less than 0.4 mm. The Houston’s coefficient of reliability was 98.18% to 99.94%, indicating a high level of reliability.

### Statistical analyses

The collected data were analyzed using the IBM SPSS Statistics 21.0 with paired-sample *t* test to compare the differences between pre- and post-treatment within each group. ANOVA test was used to evaluate the differences among the three groups pre- and post-treatment, respectively, and LSD *post hoc* analyses were applied to test the differences in between. The relationship between the BOP and the skeletal/dental patterns was assessed by means of the coefficient of correlation. The comparison and correlation between BOP and FOP were analyzed with paired-sample *t* test and coefficient of correlation, respectively. Null hypotheses were that the measurements had no differences between pre- and post-treatment within each group, that there were no differences among the three groups, and that there were no differences between BOP and FOP inclinations. The statistically significant level was set at *p <* 0.05.

## Results

The angular and linear measurements from pre-treatment and post-treatment cephalograms are listed in Table 3, and the results of the statistical analyses are presented in Table 4.

**Table 3:** **Descriptive statistics of inclination of occlusal plane and other skeletal/dental variables pre- and post- treatment**

Measurements	Skeletal class I (***n***= 30)			Skeletal class II (***n***= 30)			Skeletal class III (***n***= 30)		
	Mean ± SD			Mean ± SD			Mean ± SD		
	Pre***-***	Post***-***	Differences	Pre-	Post-	Differences	Pre-	Post-	Differences
Skeletal frame									
SNA°^a^	81.99 ± 2.85	81.54 ± 3.72	−0.46 ± 1.57	81.33 ± 2.88	81.30 ± 2.85	−0.76 ± 1.15	78.33 ± 3.67	78.74 ± 3.46	0.42 ± 1.21
SNB°	79.32 ± 2.95	79.36 ± 3.81	0.04 ± 1.35	76.60 ± 3.06	77.16 ± 2.67	0.56 ± 1.40	79.45 ± 3.38	79.66 ± 3.92	0.22 ± 1.38
ANB°	2.68 ± 0.64	2.18 ± 0.87	−0.49 ± 0.99	5.44 ± 0.88	4.15 ± 1.24	−1.29 ± 1.11	−1.11 ± 0.96	−0.93 ± 1.10	0.18 ± 1.22
MP-SN°	31.52 ± 4.04	32.09 ± 4.78	0.57 ± 1.55	34.59 ± 4.71	34.75 ± 4.59	0.16 ± 1.88	32.04 ± 6.00	32.07 ± 6.20	0.03 ± 1.48
Occlusal plane									
BOP-SN°	14.57 ± 3.82	15.29 ± 4.29	0.72 ± 2.59	17.34 ± 4.43	18.85 ± 4.12	1.51 ± 3.11	14.41 ± 4.29	14.67 ± 4.72	0.26 ± 2.32
FOP-SN°	14.70 ± 4.10	14.00 ± 4.68	−0.69 ± 3.61	17.36 ± 4.58	17.96 ± 4.44	0.60 ± 2.96	14.65 ± 4.47	14.16 ± 4.78	−0.49 ± 2.98
MxOP-MnOP°	8.10 ± 2.44	3.50 ± 1.43	−4.60 ± 2.82	8.77 ± 3.30	3.70 ± 1.08	−5.07 ± 3.01	5.69 ± 3.22	3.83 ± 1.38	−1.86 ± 3.29
Dental									
Overbite (mm)	3.79 ± 1.31	1.63 ± 0.70	−2.16 ± 1.46	4.30 ± 1.70	1.73 ± 0.63	−2.57 ± 1.51	2.34 ± 1.66	1.67 ± 0.68	−0.67 ± 1.71
Overjet (mm)	4.01 ± 1.28	2.76 ± 0.58	−1.25 ± 1.30	4.81 ± 1.86	2.63 ± 0.57	−2.18 ± 1.96	2.54 ± 1.37	2.53 ± 0.66	0.24 ± 1.39
Maxillary dentoalveolar									
U1-SN°	103.02 ± 6.41	108.36 ± 5.91	5.33 ± 7.72	99.53 ± 6.19	105.68 ± 4.74	6.16 ± 7.88	105.23 ± 5.07	109.73 ± 5.98	4.50 ± 5.65
U1-PP (mm)	26.29 ± 2.59	26.70 ± 2.58	0.41 ± 1.54	26.66 ± 3.11	27.31 ± 3.40	0.65 ± 1.21	25.49 ± 3.05	26.42 ± 3.42	0.93 ± 1.51
U6-PP (mm)	19.98 ± 2.22	21.30 ± 2.39	1.32 ± 1.49	19.56 ± 2.64	20.83 ± 2.55	1.28 ± 1.20	20.17 ± 2.55	21.67 ± 2.65	1.50 ± 1.33
Mandibular dentoalveolar									
L1-SN°	54.79 ± 6.33	51.20 ± 5.83	−3.60 ± 6.26	49.38 ± 6.96	44.65 ± 5.26	−4.73 ± 4.56	59.94 ± 6.69	59.10 ± 6.62	−0.84 ± 5.09
L1-MP°	93.68 ± 6.82	96.71 ± 5.31	3.03 ± 6.77	96.04 ± 6.37	100.43 ± 6.06	4.40 ± 4.31	88.02 ± 5.88	88.89 ± 5.79	0.87 ± 5.21
L1-MP (mm)	37.30 ± 3.08	38.78 ± 3.27	1.48 ± 2.28	38.21 ± 2.79	39.38 ± 3.70	1.16 ± 2.47	36.23 ± 3.13	37.70 ± 3.38	1.47 ± 1.09
L6-MP (mm)	27.52 ± 2.30	29.91 ± 2.47	2.39 ± 1.81	27.86 ± 2.58	30.69 ± 2.61	2.82 ± 1.75	26.81 ± 2.46	28.79 ± 2.40	1.98 ± 1.01

^a^ “°” represents the unit of angle measurement “degree”.

**Table 4:** **Comparison of inclination of occlusal planes and other skeletal/dental variables within and between groups**

Measurements	Within groups (***p***value)			Between groups (***p***value)					
	Pre- vs. post- treatment			Pre-treatment			Post-treatment		
	Skeletal I	Skeletal II	Skeletal III	I vs. II	I vs. III	II vs. III	I vs. II	I vs. III	II vs. III
Skeletal frame									
SNA°^a^	0.121	0.001**	0.071	0.944	0.000***	0.000***	0.781	0.002**	0.004**
SNB°	0.867	0.038*	0.401	0.001**	0.876	0.001**	0.017*	0.742	0.007**
ANB°	0.012*	0.000***	0.589	0.000***	0.000***	0.000***	0.000***	0.000***	0.000***
MP-SN°	0.054	0.648	0.907	0.019*	0.687	0.051	NS	NS	NS
Occlusal plane									
BOP-SN°	0.138	0.013*	0.539	0.012*	0.882	0.008**	0.002**	0.586	0.000***
FOP-SN°	0.302	0.279	0.372	0.021*	0.967	0.019*	0.001**	0.898	0.002**
MxOP-MnOP°	0.000***	0.000***	0.013*	0.384	0.003**	0.000***	NS	NS	NS
Dental									
Overbite (mm)	0.000***	0.000***	0.063	0.207	0.000***	0.000***	NS	NS	NS
Overjet (mm)	0.000***	0.000***	0.434	0.043*	0.000***	0.000***	NS	NS	NS
Maxillary dentoalveolar									
U1-SN°	0.001**	0.000***	0.000***	0.025*	0.152	0.000***	0.067	0.343	0.006**
U1-PP (mm)	0.154	0.006**	0.002**	NS	NS	NS	NS	NS	NS
U6-PP (mm)	0.000***	0.000***	0.000***	NS	NS	NS	NS	NS	NS
Mandibular dentoalveolar									
L1-SN°	0.004**	0.000***	0.375	0.004**	0.005**	0.000***	0.000***	0.000***	0.000***
L1-MP°	0.019*	0.000***	0.366	0.155	0.001**	0.000***	0.014*	0.000***	0.000***
L1-MP (mm)	0.001**	0.015*	0.000***	0.240	0.172	0.012*	NS	NS	NS
L6-MP (mm)	0.000***	0.000***	0.000***	NS	NS	NS	0.233	0.086	0.004**

^a^ “°” represents the unit of angle measurement “degree”; *p < 0.05; **p < 0.01; ***p < 0.001; *NS*, not statistically significant.

### Within groups

In class II subjects, the SNA angle decreased 0.75° (*p <* 0.01) with the SNB angle increasing 0.56° (*p <* 0.05), and the angle of ANB decreased 1.29° (*p <* 0.001), which indicated therapeutic responses as well as skeletal maturity effects, and facial balance improvement was obtained in this group. The changes of SNA angle and SNB angle were not statistically significant (*p >* 0.05) in class I and class III groups, while ANB angle has 0.49° decrease in class I group which is statistically significant (*p <* 0.05). The angle between the mandibular plane and SN plane remained the same after treatment in the three groups (*p >* 0.05).

The inclination of BOP increased by a mean of 1.51° in skeletal class II group, and the changes were statistically significant (*p <* 0.05). There were no statistically significant differences in BOP-SN angle of the skeletal class I and skeletal class III groups between pre- and post-treatment. The FOP-SN angle showed stability (*p >* 0.05) compared the post- with the pre-treatment in all of the three groups. The angle between MxOP and MnOP decreased by 4.60°, 5.07°, and 1.86°, respectively, in the three groups; and the results were statistically significant (*p <* 0.05).

The results of correlation between BOP and FOP showed that the inclination of FOP was closely related to the inclination of BOP (*p <* 0.001) in each group (Table 5). Comparison between FOP and BOP revealed a significant discrepancy that the inclination of FOP was greater than that of BOP in each group but only showed statistical significance in class III patients (0.24°, *p <* 0.001) before treatment. After orthodontic therapy, the differences between FOP and BOP emerged in the class I and class II groups with the angles of 1.29° and 0.90° (*p <* 0.05), respectively, while no significant divergence was detected in the class III group (*p >* 0.05) (Table 6), which might be connected with the relative rotation between FOP and BOP.

**Table 5:** **Correlation between BOP and FOP in each skeletal pattern**

(BOP-SN°)-(FOP-SN°)^a^		Skeletal class I (***n***= 30)	Skeletal class II (***n***= 30)	Skeletal class III (***n***= 30)
Pre-treatment	Correlation	0.769***	0.872***	0.838***
	*p* value	0.000	0.000	0.000
Post-treatment	Correlation	0.912***	0.920***	0.948***
	*p* value	0.000	0.000	0.000

^a^ “°” represents the unit of angle measurement “degree”; ***p < 0.001.

**Table 6:** **Comparison between BOP and FOP in each skeletal pattern**

(BOP-SN°)-(FOP-SN°)^a^		Skeletal class I (***n***= 30)	Skeletal class II (***n***= 30)	Skeletal class III (***n***= 30)
Pre-treatment	Mean ± SD	−0.13 ± 2.71	−0.02 ± 2.29	−0.24 ± 3.44
	*p* value	0.799	0.968	0.000***
Post-treatment	Mean ± SD	1.29 ± 1.93	0.90 ± 1.74	0.52 ± 1.53
	*p* value	0.017*	0.009**	0.076

^a^ “°” represents the unit of angle measurement “degree”; *p < 0.05; **p < 0.01; ***p < 0.001.

In skeletal class I and class II groups, overbite, overjet, the inclination of U1 and L1 (U1-SN, L1-SN, L1-MP), and the vertical height of U1, U6, L1, and L6 (U1-PP, U6-PP, L1-MP, L6-MP) changed statistically significantly (*p <* 0.05) after treatment except for U1-PP in class I group (*p >* 0.05). The overbite, overjet, and the inclination of L1 (L1-SN, L1-MP) found to be stable (*p >* 0.05) in the skeletal class III group with other parameters changed significantly (*p <* 0.05).

### Between groups

Through the comparison of the pre-treatment measurements of class II and class III groups with class I group with normal malocclusions, the results showed that skeletal class II malocclusions were mainly due to mandibular retrusion (smaller SNB, larger mandibular plane angle, compared to class I, *p <* 0.05) with a normal SNA angle (*p >* 0.05), while results showed the opposite in skeletal class III group with maxillary retrusion (smaller SNA compare to class I (*p <* 0.05), normal SNB). The same trend of the antero-posterior position of the maxilla and mandible was found even after orthodontic treatment. No statistical differences were detected from the mandibular plane angle among the three groups after treatment.

The inclination of BOP and FOP were statistically significantly steeper in class II subjects compared to the other two groups, both before and after treatment (*p <* 0.05), while showing flat in both skeletal class I and class III groups. There were no statistically significant differences in the inclination of BOP (and FOP) between the class I and class III groups (*p >* 0.05) before and after treatment. The angles between MxOP and MnOP were observed significantly smaller in the class III group than those in class I and class II subjects (*p <* 0.05) before treatment, while these angles were significantly decreased (*p <* 0.05) in all of the three groups and showed no differences (*p >* 0.05) after orthodontic treatment with the improvement of the relationship between upper and lower incisors. The overbite and overjet were corrected to normal after treatment (*p >* 0.05), which showed significant differences among these three groups (*p <* 0.05) before treatment, except for overbite between class I and class II subjects (*p >* 0.05).

### Correlation coefficient

In skeletal class II group, we found that BOP increased significantly (*p <* 0.05) after orthodontic treatment; coefficient of correlation was taken to evaluate the correlation coefficient between the inclination changes of BOP and the changes of skeletal/dental patterns in this group after treatment (Table 7).

**Table 7:** **Correlation between changes of BOP inclination and Skeletal/Dental patterns in class II group after treatment**

		SNA	SNB	ANB	MP-SN	Overbite (mm)	Overjet (mm)	U1-SN	U1-PP (mm)	U6-PP (mm)	L1-SN	L1-MP	L1-MP (mm)	L6-MP (mm)
(BOP-SN°)^a^	Correlation	−0.547**	−0.471**	0.020	0.431*	−0.417*	−0.434*	−0.443*	0.291	−0.576**	−0.474**	0.278	−0.614***	−0.037
	*p* value	0.002	0.009	0.916	0.017	0.022	0.017	0.014	0.119	0.001	0.008	0.136	0.000	0.844

^a^ “°” represents the unit of angle measurement “degree”; *p < 0.05; **p < 0.01; ***p < 0.001.

The results showed that the inclination of BOP was significantly related to the mandibular plane (SN-MP°, 0.431). In other words, although the change of SN-MP angle due to treatment is not significant (*p >* 0.05), BOP is related to the posterior rotation of the mandible.

The correlation analysis also showed significant correlation with the overbite (−0.417), overjet (−0.434), U1-SN angle (−0.443), L1-SN angle (−0.474), U6-PP distance (−0.576), and L1-MP distance (−0.614). This finding suggested that the increased inclination of BOP in the class II group was closely associated with the proclination of U1 and L1 and the increased vertical height of U6 and L1 (Table 3).

## Discussion

In this study, we attempted to determine if there was any difference in the inclination of OP in subjects with three types of skeletal malocclusion treated with a non-extraction orthodontic protocol. Skeletal and dental changes were evaluated by cephalometric analysis.

In the present study, the average ages of each group when the treatment started were 13.75 ± 1.54, 13.03 ± 1.41, and 13.63 ± 1.44 years, respectively. So we still need to consider the influence of residual growth and skeletal maturity, which might take place as well as therapeutic effects. Complicated by growth and maturational changes within the cranial structures that happen concurrently, the treatment induced changes of the OP in adolescents are not easy to determine [3, 23]. Although group composition, such as gender, pre-treatment ages, and duration of treatment in each group were similar in this study (Table 1), the growth potential among each group may or may not be the same. Bishara [24] stated that the differences in craniofacial measures were established early in life, and the growth trends in class II and class I subjects appeared to be essentially similar thereafter. Other studies confirmed that the features of class II dentoskeletal disharmony established on the pre-pubertal stage of development but associated with significant deficiencies in the growth of the mandible than those in class I subjects [23, 25, 26]. The skeletal imbalance in class III malocclusion is also established early in life, and the disharmony becomes more pronounced in the majority of patients during the pubertal peak and continues until skeletal maturation is complete [27, 28].

Therefore, in the present study, the angles of SNA, SNB, ANB, and SN-MP were taken into consideration to evaluate the combined effect of both growth and treatment on maxillary and mandibular antero-posterior position. The comparison of these angles among the three groups before treatment revealed that the skeletal class II individuals were mainly due to mandibular retrusion, whereas retrusive maxilla was the significant cause of class III malocclusions, which is consistent with the report of Ellis and McNamara [29]. Comparing the difference within each group, significant changes of SNA, SNB angulation (*p <* 0.05) were found in class II individuals, with the ANB angle decreased 1.29° after orthodontic treatment, which corrected the skeletal disharmony of maxilla and mandible. No significant changes of SNA angle or SNB angle were observed in class I and class III groups (*p >* 0.05), while the ANB angle decreased significantly in class I group (*p <* 0.05). The changes of ANB angle in class I subjects agreed with normal growth conditions, by observing 28 untreated class I malocclusion subjects from 6 to 20 years of age, in which studies showed that the ANB angle decreased continuously until age 14 years [30]. Ochoa and Nanda [30] also stated that the maxillomandibular convergence increased with age as described by decreasing in MP-SN angle, but in the present study, we found that the angle between the mandibular plane and SN plane remained stable after treatment in all of the three groups (*p >* 0.05).

However, in studies which showed that OP itself experiences physiological growth-related changes, Chang et al. [21] found that the occlusal plane tended to rotate forward due to growth in patients with normal occlusion, and Vukusic et al. [31] also described that the angle between OP and cranial base decreased from 19.75° to 16.44° in patients between 10 and 18 years old. The study also found that the changes seem to be different among different skeletal configurations [2]. Thus, the effect of growth on the change of the occlusal plane angle between each group in this study needs to be taken into consideration.

In this investigation, we found that the inclinations of both BOP and FOP in skeletal class II subjects were significantly different from that in skeletal class I and class III groups before treatment. The BOP and FOP were steep in the skeletal class II group, while in skeletal class I and class III groups they were flat. Downs stated the same detection in his study of facial patterns [13]. This finding also agrees with Tanaka and Sato [2], who stated that during a longitudinal investigation of 102 orthodontically untreated white patients, statistical significance was reached at 12 to 14 years among class II vs. class I and class III, and they proposed OP as a determinant for malocclusion. It is noteworthy that this distinction exists even after orthodontic treatment.

The occlusal plane forms following the establishment of occlusion. In a growing facial skeleton, the position of OP is determined largely by the vertical growth of the maxillary teeth, and the inclination of the OP is determined largely by the growth of the dentoalveolar bone [2]. Besides the growing factors mentioned above, the maintenance or changing of the OP during orthodontic treatment depends on mesial molar movement, vertical control of the maxillary and mandibular molars, and extrusion and intrusion of incisors [1, 20]. The amount of mesial molar movement is often very little in non-extraction case, so only the other two factors were considered.

An attempt to find out whether OP inclination alternated with treatment, related measurements were conducted. The results revealed that the angle measured between BOP and SN plane is statistically significantly increased in skeletal class II group after orthodontic treatment by an average of 1.51**°** (*p =* 0.013), while no statistically significant differences were detected in class I and class III groups. In agreement with Fushima et al. [7], we also found that the class II malocclusions have steep BOP and FOP before treatment. Orthodontic treatment improved the occlusal relationship of class II patients, but it seems that the BOP becomes steeper with the clockwise rotation. An explanation for such a change could be the extrusion of molars and incisor (Table 3) by treatment mechanics of skeletal class II patients and residual vertical growth of the patients [1, 3, 23, 32].

The class II elastics that are often used to correct a class II malocclusion, so-called class II mechanics, could cause the downward and backward rotation of the OP due to mandibular molar extrusion and maxillary incisor extrusion [1, 32], consequently, increased the angle of the OP with SN plane [33]. Zimmer et al. [19] also observed several significant changes in the occlusal plane inclination due to oppositely guided intermaxillary elastics, the induced shift with class II elastics was clockwise, while class III elastics was counterclockwise. The patients included in this study had growth potential, so the change of OP inclination is naturally growth-related changes, and the use of class II elastics completely eliminate typical growth-induced decreases in inclination [19]. But in contrast to Zimmer’s report, in the class III group, the BOP inclination change after treatment was not statistically significant in our study.

The side effect of class II elastics could also change the vertical dimension of patients [1]. Anchorage preparation in class II malocclusions could enhance vertical alveolar growth in the mandibular arch and a compensating condylar growth [1]; along with the molar extrusion and the growth of the anterior facial, the mandibular plane angle can be maintained, but the face may become longer. This could explain that in the present study, the angle between MP-SN remained stable after treatment, even in skeletal class II group that the inclination of BOP statistically significantly increased due to treatment.

In this study, correlation analysis was used to study the relationship between the inclination changes of BOP and the changes of skeletal/dental patterns in skeletal class II group. Although the change of MP-SN angle due to treatment is not statistically significant (*p >* 0.05), we found that there was a correlation between the inclination of mandibular plane and the inclination of the occlusal plane. We also found that the increased inclination of BOP was closely related to the forward inclination of U1 and L1 and the eruption of U6 and L1 (Table 3). Although the average vertical eruption of U6 (1.28 ± 1.20 mm) is greater than L1 eruption (1.16 ± 2.47 mm), with the L1 inclined forward 4.73° (L1-SN), the occlusal plane rotated downward 1.51°.

The BOP and FOP landmarks used in the present investigation are different from those used by Downs [13] and Braun et al. [14], respectively. Instead of using the midpoint of mesio-buccal cusps of the upper and lower molars as posterior landmark, we employed the point bisecting the U6 occlusal and L6 occlusal, which is provided by the Dolphin Imaging software digitized function under the name ‘Occlusal Plane Distal.’ This may cause some differences compared with other experiments. During the tracing process, we found that the correct identification of the landmarks ‘upper first bicuspid’ and ‘lower first bicuspid’ was difficult, especially in malpositioned teeth, and this affected the repeatability of FOP giving a measurement error of 1.0°. The inclination of FOP revealed the same tendency as BOP before treatment, which was steep in class II individuals and flat in both skeletal class I and class III subjects. The same trend continued after orthodontic treatment.

Although the inclination of FOP was closely related to the inclination of BOP, we found a significant discrepancy of the inclination of FOP and BOP within each group. The inclination of FOP was greater than that of BOP in each group but only showed statistically significant differences in class III subjects (*p <* 0.001) before orthodontic treatment. After orthodontic therapy, only BOP inclination increased statistically significantly in the class II group, but due to the relative rotation between FOP and BOP, distinction appeared in the class I and class II groups while no significant divergence was detected in the class III group.

The current research also found that there were no statistically significant changes in the FOP inclination in orthodontically treated patients with the three skeletal patterns, which could be an important factor in post-treatment stability. The FOP represents a structural limitation of mandibular motion, and all masticatory forces are focused on this plane and intimately related to it. The change of muscular environment is cited as a cause for relapse; if the inclination of functional OP changed remarkably, the OP might revert to its original position after treatment [33].

This study provides a predictive framework for the potential changes of the inclination of OP during orthodontic treatment. Further studies are required for the change of occlusal plane inclination of patients in extraction-based therapy as well as surgical cases. It has been proposed that in class II patients, those cases exhibiting the greatest growth during treatment exhibited the least change in the inclination of the occlusal plane while showed the greatest tendency to return to the original inclination; conversely, those cases exhibiting the least growth during treatment exhibited the greatest change in the occlusal plane and showed less tendency to return to the original inclination [33]. It would be of interest to study the influence of occlusal plane inclination changes on the relapse of orthodontically treated patients in the long term.

## Conclusions

In this study, it was found that the inclinations of BOP and FOP were statistically significantly steeper in class II subjects compared to the other two groups both before and after treatment. The results also revealed that the BOP angle increased in all of the three groups but only had statistically significant differences in skeletal class II patients with an increase of 1.51°. We also found that the BOP was a more reproducible reference plane compared with FOP during cephalometric tracing process, while FOP showed stability in orthodontically treated patients with all three skeletal patterns.

## Consent

Written informed consent was obtained from the patient for the publication of this report and any accompanying images.

## Authors’ original submitted files for images

Below are the links to the authors’ original submitted files for images. Authors’ original file for figure 1

## Abbreviations

BOP: Bisected occlusal plane:
FOP: Functional occlusal plane:
MnOP: Mandibular occlusal plane:
MP: Mandibular plane:
MxOP: Maxillary occlusal plane:
OP: Occlusal plane:
PP: Palatal plane:
SN: Sella-nasion plane:
