# Supplementary figures and images for: Changes of occlusal plane inclination after orthodontic treatment in different dentoskeletal frames
Source: Prog Orthod. 2014 Jun 25;15:41. doi: 10.1186/s40510-014-0041-1 (PMC4884032; doi:10.1186/s40510-014-0041-1)

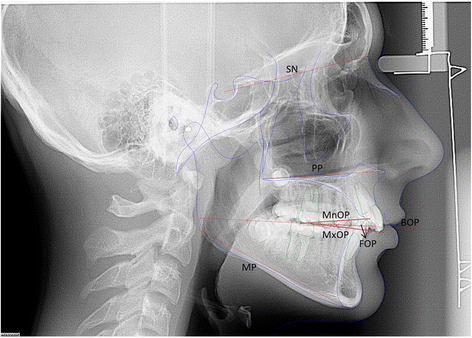

Supplement: Supplementary file 1 — Authors’ original file for figure 1 [file 40510_2014_41_MOESM1_ESM.gif]
